# Achieving the SDGs in the European Region

**DOI:** 10.1093/eurpub/ckaa025

**Published:** 2020-05-11

**Authors:** Iveta Nagyova, Martin McKee, Maaike Droogers

**Affiliations:** c1 European Public Health Association (EUPHA), Utrecht, Netherlands; c2 Department of Social and Behavioural Medicine, PJ Safarik University, Kosice, Slovak Republic; c3 Centre for Global Chronic Conditions, London School of Hygiene & Tropical Medicine, London, UK; c4 European Observatory on Health Systems and Policies, Brussels, Belgium

Abbreviations have their uses but, especially when they gain widespread usage, there is a risk that we forget what they actually stand for. This supplement is about the SDGs.^1^ The G is for Goals. The 17 goals have been agreed by each of the 53 member states of the World Health Organization (WHO) European Region and by the European Union. Goals are things to be achieved, and by a particular time, in this case 2030. Each goal has a series of targets and progress to achieving each target is measured by one or more indicators. Governments have committed to these goals. But they will only be achieved if those governments develop strategies and implement policies that can make things happen. Almost five years into the process, from the 2015 baseline, it is reasonable to expect many of these measures to be in place and to have evidence that progress is being made. And it is essential that the politicians are held accountable for their actions or, in some cases, inactions.

D is for Development. The SDGs cover a wide range of issues, all of which contribute to making the world a better place for everyone. They include the basics of life, such as access to food, water, shelter and security. They promote progress, through economic development and essential infrastructure. And they recognize the mutually reinforcing relationships between health, economic growth and social cohesion.^2^ They have, at their core, equity, between rich and poor and men and women, making clear the point that we are talking of development for the many, not the few.

S is for Sustainable. History is littered with examples of civilizations that have depleted or destroyed their natural resources, leaving only the relics of their past achievements. Today, tourists come from far and wide to see those relics, such as the statues on Easter Island or the Mayan temples in central America, but the political and economic structures from which they emerged are long gone.^3^ As the Brundtland Commission reminded us, the challenge is to find a way of ‘meeting the needs of the present without compromising the ability of future generations to meet their own needs.’^4^

This supplement is part of the process of ensuring accountability. It asks what we, in Europe, have achieved and what else must we do.

In the first paper, Bettina Menne and colleagues offer a framework for action to accelerate progress towards the SDGs. In a process lasting 3 years they began by identifying tools that held the potential to take forward the SDG agenda, describing the governance arrangements that governments had put in place to progress towards the SDGs, and examined what they had achieved. They then engaged in a wide consultation with stakeholders from governments, international agencies, civil society and academia. They found evidence of considerable activity but most of it was poorly co-ordinated. It was not clear that success was likely without a step change in pace and intensity of action.

Their framework has the memorable title E4A. E is for Engage. This means working across sectors to ensure that everyone who should be involved is involved. It includes raising awareness of what needs to be done, and by whom. And it challenges rigid institutional boundaries. Then come the 4As, which are the building blocks of the process of engagement. Assess determines the distance to be travelled to achieve the SDGs and the factors that will facilitate or inhibit progress, Align involves harmonizing policies and processes across sectors. Accelerate means identifying policies and programmes that can increase the pace of change and support innovation. Account involves ensuring that governments deliver on their commitments.

The second paper, by Maaike Droogers and colleagues, reports on progress towards the health-related SDGs in Europe. There are some important challenges. Despite impressive progress in some areas, Europe is not performing well on alcohol and tobacco, suicide and overweight among children. Each is examined in turn, examining the direction of travel of current policies, continuing challenges, and ways forward. Written from the perspective of the European Public Health Association (EUPHA), it emphasizes the potential contribution of civil society organizations.

The third paper, on the environment and health, is by Francesca Racioppi and colleagues. It recalls how environment and health are linked in several SDGs, from the traditional aspects of environmental health, such as clean water and sanitation, through to new ways of thinking about the built environment, as in SDG 11 (sustainable cities and communities), where there is now a very innovative thinking about how urban planning can promote health.

In the fourth paper, Giuseppe Grosso and colleagues remind us of the role that food and nutrition play in many of the SDGs. This extends beyond SDG 2, the elimination of hunger, to good health (SDG 3), responsible consumption (SDG 12) and life on land (SDG 15) and below water (SDG 14). They highlight the major contribution of appropriate food policies to SDG 13 (climate action), given the importance of agriculture as a source of greenhouse gases. But progress will only be made if we are willing to accept a major change to our diets, with a shift to plant-based foods.

The fifth paper, by Tatjana Buzeti and colleagues, reminds us that equity is at the core of the SDGs. They urge European governments to build on the WHO European Equity Status Report Initiative. This is fully aligned with the SDGs, identifying the five conditions that must be in place if we are to ensure healthy and prosperous lives for all. These are universal access to health services, income security and social protection, safe and decent living conditions, inclusive measures to build human and social capital and decent, non-discriminatory working conditions. This initiative contributes to the Assess component of the E4A approach as it collates detailed data on where the member states are in creating these five conditions. It also addresses the Align component, identifying the policies that offer the greatest scope to make progress towards the SDGs. But it also argues that we need to go beyond the SDGs, to ensure that an equity perspective is included in all of them, and not just those where it already exists. This can only happen, however, with enhanced data systems that include measures of equity, thereby making visible the inequities that are too easily overlooked.

A sixth paper, by Selina Rajan and colleagues, examines the role of health systems in achieving the SDG target 3.8, to achieve universal health coverage. It begins by examining the challenges of operationalizing the associated indicators, coverage of essential health services and financial protection. It describes the progress that has been made so far, by the WHO^5^ and the Global Burden of Disease programme^6^ in creating measures of effective coverage, but highlights the challenge of how to include the needs of disadvantaged populations. These are often excluded from existing data collection, inadvertently or by design. In some cases, they are the victims of discriminatory policies, a growing problem in some countries that have adopted a hostile environment for groups such as undocumented migrants. There are also many gaps in our information on financial protection, although work by the WHO Barcelona office is making an increasingly important contribution to our understanding.^7^

The seventh paper, by Dineke Zeegers Paget and David Patterson, recalls that health is a human right and examines the way in which it can be upheld by the law. It begins by reviewing some of the traditional ways in which the law has been used, including action on threats to health, such as tobacco, and on measures to tackle discrimination. However, the authors identify a new series of challenges where, they argue, the law can be an ally of public health. These include legal measures to ensure that advances in technology and information are harnessed for the public good rather than undermining, how to uphold the right to health care in times of austerity, and legal measures to address the commercial determinants of health.

The eighth paper, by Natasha Azzopardi-Muscat and colleagues, examines the political processes that will be required to achieve the SDGs, and in particular the challenges that are created by the distribution of power within society.

The ninth paper, by Karin Sipido and Iveta Nagyova, examines the role of research in achieving the SDGs. They begin by pointing to evidence on the return of investment in health research and then examine the contribution that research has made to the remarkable decline in cardiovascular disease in recent decades. However, as they note, despite many achievements in expanding our understanding of this condition and identifying new treatments, there has been much less success in implementing this new knowledge. Hence, they argue for a much greater investment in knowledge translation and getting evidence into policy.

The supplement closes with three case studies describing how different parts of Europe have taken forward initiatives related to the SDGs. The Welsh government has enacted a Well-Being of Future Generations Act, which places a duty on national and local public authorities to contribute to the economic, social, environmental and cultural well-being of the country. The authors describe how the legislation embeds sustainable development in all policies, providing an example from which others can learn. Montenegro describes how WHO can catalyze action, in this case through its small countries initiative, and how the health ministry can convene stakeholders from a wide range of sectors to collaborate, in this case to address the problem of water and sanitation. The final case studies from Estonia, a country that historically had a very high rate of alcohol-related disease, but which has now achieved substantial improvements, describes the process by which intersectoral action was achieved.

The countries of the European Region have only a decade to achieve the SDGs. There has been considerable progress, but there is much still to do, and Europe as a whole, and individual member states, will not hit the targets with business as usual. The E4A approach offers a novel way of moving forward, calling on governments to engage widely, with everyone who can make a difference. Then they must assess where they are now and whether they are on track to achieve the SDG targets. Several of the papers in this supplement, such as that of health systems, should help them as they design the necessary monitoring systems. Success will depend on governments’ ability to align their policies and processes across sectors and levels of government in ways that support progress towards the SDGs, but this is not enough. They must identify ways to accelerate activity, recognizing that 2030 is not far away. Finally, there must be systems that allow governments to be held to account for the commitments they have made in the SDGs.

*Conflicts of interest*: None declared.
